# PLX3397-Induced Microglial Ablation Alters Adipose Tissue Accumulation in a Male–Female-Dependent Manner Under High-Energy-Diet Feeding

**DOI:** 10.3390/nu17213445

**Published:** 2025-10-31

**Authors:** Flynn P. O’Connell, Andras Hajnal, Patricia M. Di Lorenzo, Krzysztof Czaja

**Affiliations:** 1 Department of Psychology, Binghamton University, Binghamton, NY 13902, USA; oconnell@binghamton.edu (F.P.O.); diloren@binghamton.edu (P.M.D.L.); 2 Department of Neural and Behavioral Sciences, College of Medicine, The Pennsylvania State University, Hershey, PA 17033, USA; axh40@psu.edu; 3 Department of Biomedical Science, School of Veterinary Medicine, Ross University, Basseterre P.O. Box 334, Saint Kitts and Nevis

**Keywords:** microglia ablation, dietary obesity, taste preference, Pexidartinib, hypothalamus, nucleus tractus solitarius, PLX 3397, high energy diet

## Abstract

Background: Diet-induced obesity (DIO) is increasingly linked to microglial proliferation in the central nervous system, yet the causal role of microglia in metabolic and behavioral changes remains unclear. Methods: Here, we investigated the effects of microglial suppression using the CSF-1R antagonist PLX 3397 (Pexidartinib; PLX) on body weight, adiposity, and sucrose preference in lean and DIO male and female rats. Microglial activation was quantified in the hypothalamus and nucleus tractus solitarius (NTS). Results: PLX administered during initial high-energy-diet (HED) exposure produced sex-specific effects: body weight increased in males but decreased in females. In male DIO rats, PLX+HED reduced body fat percentage without altering total weight. PLX treatment did not significantly alter body weight, food intake, or glucose tolerance in females. Hypothalamic microglial suppression was more extensive in males, whereas NTS suppression was similar across sexes. PLX also reversed HED-induced reductions in low-concentration sucrose preference in males. Substantial individual variability was observed in both susceptibility to DIO and responsiveness to PLX. Conclusions: These findings reveal a clear sexual dimorphism in microglial responses to HED, with females showing relative protection and males’ greater vulnerability. Overall, the results underscore the importance of accounting for sex differences in the design and application of microglia-targeted interventions.

## 1. Introduction

Obesity is associated with serious, life-threatening health risks and has seen increased prevalence in the United States since 1999 [1]. Obesity is an inflammatory disease; excess adiposity promotes a proinflammatory state in which peripheral and central macrophages contribute to a state of chronic, low-grade inflammation. Microglia are the resident immune cells in the central nervous system (CNS) and maintain local populations through self-renewal, independent of bloodborne macrophages [2,3]. Activated microglia detect and eliminate pathogens and have recently been shown to mediate obesity susceptibility as regulators of the metabolic consequences of high energy diet (HED) consumption [4]. Prolonged microglial activation correlates with disruptions in satiety signaling, metabolic homeostasis and subsequent weight gain [5,6,7,8,9,10,11]. Evidence suggests microglia also regulate consummatory behavior and body weight under normal dietary conditions [5]. Thus, microglia represent a central therapeutic target for obesity and branching comorbidities.

Previous studies examining microglial impact on metabolism almost exclusively assessed microglial ablation immediately after the introduction of a HED. There is minimal real-world therapeutic efficacy in this approach as many of the primary and secondary metabolic consequences of obesity require decades to develop [12]. The effects of microglial ablation under different dietary conditions remain unclear. Thus, we sought to elucidate the impact of microglial ablation under normal dietary circumstances, immediately after initiation of HED exposure, and following prolonged HED exposure. We pharmacologically eliminated microglia using dietary Pexidartinib (PLX3397; PLX). PLX is a CSF-1R antagonist that prevents monocyte/macrophage proliferation and differentiation, thus inhibiting microglial activity. To strengthen the mechanistic rationale, we also examined both hypothalamic and NTS regions to determine whether PLX-induced microglial ablation affects central circuits controlling energy balance and taste behavior through complementary but distinct pathways. Importantly, PLX eliminates 99% of microglia in the adult mouse brain without affecting surrounding monocytes and macrophages [13]. We hypothesized that long-term HED consumption would ameliorate the microglia-mediated weight loss and alterations in consummatory behavior in rats seen in previous studies using mice. Further, because obesity leads to alterations in the degree of preference for palatable stimuli, we incorporated a sucrose preference test at the end of the experiment [14,15,16,17]. In summary, by targeting microglia pharmacologically across distinct brain regions, this study aimed to determine whether region-specific inflammatory profiles underlie the divergent metabolic and behavioral consequences of chronic HED exposure.

Extensive research has described inflammation in the midbrain as crucial for homeostatic control of food intake and immune-metabolic function [6]. However, very little has been done to correlate these functions with inflammation in the hindbrain in general, and in the nucleus tractus solitarius (NTS) in particular. The NTS acts as a central gatekeeper, reciprocally connected to the periphery and upstream structures in the CNS, acting as a primary integration node for meal-related, metabolic, and inflammatory signals from the periphery [18,19,20,21,22]. Communication between the NTS and several hypothalamic nuclei promotes vagally mediated satiety and meal termination [23]. Further, HED-driven alterations to the gut–brain pathway promote long-term inflammation in the NTS [24]. Since the NTS modulates crucial meal and metabolic-information transfer to feeding centers in the midbrain and forebrain, we hypothesized that hypo-ramification of microglia in the NTS would correlate with weight loss in rats with diet-induced obesity (DIO) as well as behavioral changes related to sucrose preference.

Here, we demonstrate that dietary PLX suppressed microglial proliferation in both the NTS and hypothalamus in a strikingly sex-dependent manner. Correspondingly, effects on body composition and consummatory behavior differed between sexes, often trending in opposite directions. Notably, we provide the first evidence that microglial ablation can significantly increase body weight in males. In contrast, PLX administration partially reversed HED-induced reductions in sucrose preference in females, but not in males. The diet-dependent suppression of microglia in the NTS highlights the potential role of brainstem inflammation in the pathophysiology of DIO. These pronounced sex differences not only warrant caution in experimental interpretation but also raise important questions about the underlying mechanisms driving divergent male and female responses.

## 2. Materials and Methods

*Animal Care and Dietary Manipulations.* All procedures were in accordance with the National Institutes of Health Animal Welfare Guide and were approved by the Institutional Animal Care and Use Committee of Binghamton University (Protocol title: Temporal coding in the gustatory system of the rat; Protocol #: 795-18, 2018–2021). Male (*n* = 39) and female (*n* = 37) Sprague-Dawley rats (8 weeks old) were obtained from Taconic Labs, Inc. (Germantown, NY, USA) and allowed to acclimate to the temperature-controlled vivarium for 7 days with a 12 h light/dark cycle (lights off at 0900 h). Rats had ad libitum access to a standard chow diet (Chow) (Purina Lab Diet 5L0D, catalog 0050795, Lab Diet, St. Louis, MO, USA; 13.6% fat, 28.9% protein, 3.4% sucrose) and water during acclimation. Following acclimation, rats were, single-housed and divided into weight-matched dietary groups. For 10 weeks, rats were given ad libitum access to water and either HED (Research Diets, catalog D12451; 45% fat, 20% protein, and 21% sucrose) or Chow.

Sample size was based on prior work from our laboratory and comparable neuroinflammatory studies demonstrating sufficient statistical power (β = 0.8, α = 0.05) to detect ≥15% differences in body weight and microglial activation between treatment conditions [17,25]. A minimum of six animals per group and sex was used to ensure reliable variance estimates.

*PLX3397 (Pexidartinib).* After 10 weeks of HED or chow intake, diets were switched to plain chow, HED, or chow or HED infused with PLX3397 (PLX; MedKoo Biosciences, Morrisville, NC, USA) for an additional 6 or 7 weeks. Accordingly, the experimental groups were as follows: Chow/Chow, Chow/Chow+PLX (189.5 mg PLX/kg chow), Chow/HED, Chow/HED+PLX (300 mg PLX/kg HED), HED/HED, HED/HED+PLX. PLX concentrations were calculated to approximate the same quantity of kcal/g body weight using food-intake data from rats consuming the same HED recorded previously [17].

*Food intake, body weight, and body composition.* During the initial 10-week diet period, animal weights and food intake were measured bi-weekly. Available food was kept above 50 g as to avoid any potential rationing behaviors. Body weight measurements and food intake were not taken during preference testing to avoid excess leakage from the preference bottles. To determine the effect of diets on body adiposity, all animals underwent dual-energy X-ray absorptiometry (DXA) scans starting 4 weeks before PLX administration and subsequently every 2 weeks. All rats were DXA scanned at age-matched time points within a day of the other groups. Animals were sedated with Medetomidine HCl (Pfizer Inc., New York, NY, USA; 0.1 mg/kg, s.c.) and placed on a scanning bed where body composition was calculated by Hologic APEX Discovery A software (Hologic Apex v2.0, Bedford, MA, USA). Scan results determined both body fat and lean tissue mass. Sedation was reversed by Atipamezole (Pfizer Inc., NY, USA; 0.1 mg/kg, i.p.) upon completion of the scan.

*Two-bottle Sucrose Preference Testing.* To determine the effect of microglial depletion on preference for sweetness, we presented two concentrations of sucrose alongside water in a two-bottle, 48 h preference test. Testing began one week before animals were sacrificed. Individually housed rats were randomly split into two groups and given two bottles: one with either 0.03 M or 0.3 M sucrose solution, the other with tap water. After 24 h the position of the bottles was swapped to avoid position bias. After 48 h, concentrations were switched, tap water was re-filled and the same procedures were used for the following 48 h. Consumption was measured for each concentration before switching the side of the bottle.

Only healthy animals showing normal weight gain and no clinical signs of illness or injury during acclimation were included. Animals were excluded if they exhibited >10% weight loss, signs of infection, or technical issues during behavioral testing. One male rat from the Chow/HED+PLX group was excluded due to measurement error during sucrose preference testing.

Although randomization was not applicable during group assignment due to the need to form weight-matched dietary groups, randomization was implemented during sucrose preference testing. Environmental conditions, handling, and testing times were standardized across groups. Because rats were single-housed, social dominance effects and food competition were avoided. Circadian influences were controlled by conducting all procedures at consistent times of day. Investigators performing data collection, DXA analysis, histological quantification, and behavioral scoring were blinded to the treatment assignments. Data coding and decoding were conducted by separate personnel to ensure objectivity.

*Histology.* At the end of the experiment, rats were overdosed with Fatal Plus (sodium pentobarbital) and perfused transcardially with 4% paraformaldehyde. Brains were removed and stored in 20% sucrose in 4% paraformaldehyde.

Hindbrains were cryosectioned (Leica CM1950, Leica Biosystems, Wetzlar, Germany) at 20 μm thickness and standard immunofluorescence was used to determine microglia activation in the hindbrain. Sections were incubated overnight with a primary antibody against ionized calcium binding adaptor molecule 1 (Iba-1, Wako Cat#019-19741, RRDI: AB_839504) followed by Alexa-488 secondary antibody (Alexa 488 Donkey anti-Rabbit, Invitrogen by Thermo Fisher Scientific, Waltham, MA, USA, cat#A21206) for 2-hr to visualize microglia activation as previously described [25]. Sections were mounted in ProLong (Molecular Probes, Eugene, OR, USA) and examined under a Nikon 80-I fluorescent microscope. The area fraction of Iba1 was analyzed using Nikon Elements AR software as previously described [26,27].

Throughout the manuscript, the term “microglial ablation” refers specifically to the pharmacological depletion of Iba1^+^ microglia induced by PLX3397, rather than complete elimination of all CNS macrophages.

*Statistical analysis*. Results are expressed as mean ± SEM. Two-way repeated measures [RM] ANOVAs with appropriate corrections for multiple comparisons were used for body weight, DXA, sucrose preference and food intake data, as indicated. Statistical analysis was performed with GraphPad Prism 9.0 (GraphPad Software, Inc., San Diego, CA, USA). Differences were considered significant at *p* < 0.05.

## 3. Results

### 3.1. Effects of a HED on Body Composition

**Figure 1:**
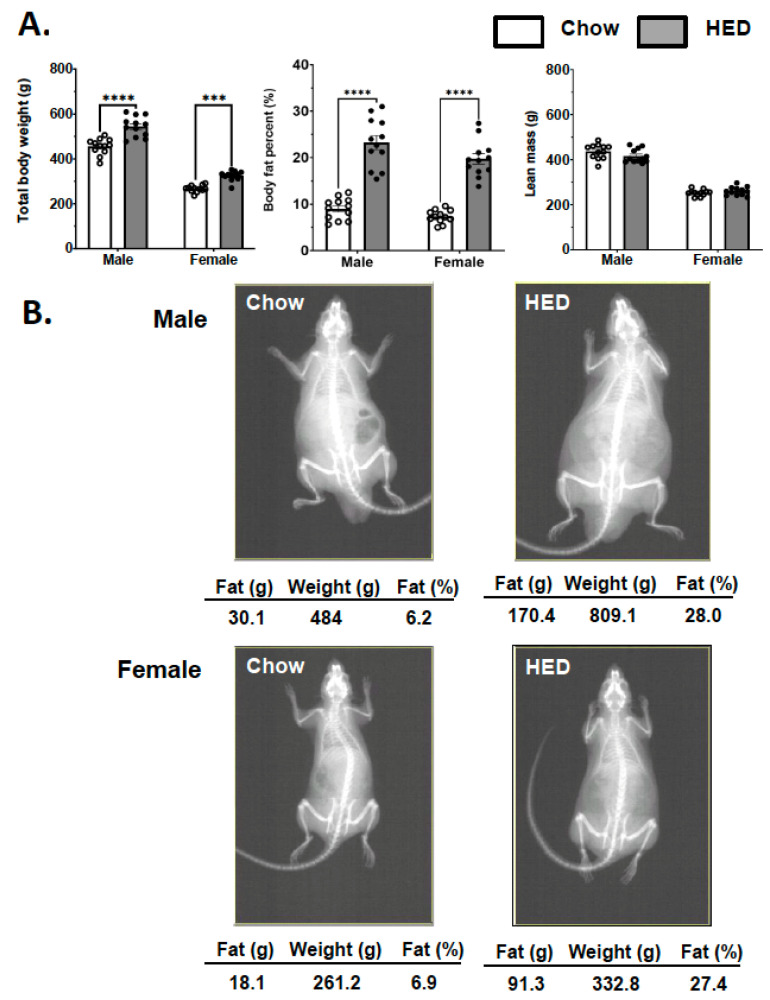
HED induces obesity via increased fat accumulation. DXA scan body composition data taken after initial 10 weeks of chow or HED. (**A**) HED produces increased body weight and percent body fat without changing lean body mass in both male and female rats. Data are expressed as mean ± SEM. *** *p* < 0.001; **** *p* < 0.0001. All statistics performed using two-way RM-ANOVA with Tukey’s correction for multiple comparisons. (**B**) Representative DXA scan images of male and female rats.

To assess the effects of exposure to a HED on body composition, we examined total body weight, percent body fat and lean body mass after 10 weeks from chow-fed males (*n* = 10) and females (*n* = 9) as well as HED-fed males (*n* = 12) and females (*n* = 12). For each measure, a two-way ANOVA was applied with sex and diet as factors. Body weight was significantly affected by gender (F (1, 44) = 497.9, *p* < 0.001) and diet (F (1, 44) = 64.44, *p* < 0.001), but there was no significant interaction (F (1, 44) = 2.88, *p* = 0.097). Both male and female (Figure 1A) rats fed a chow diet weighed significantly less than rats fed a HED (males, 455.1 ± 10.3 g for chow-fed vs. 543.9 ± 13.1 g for HED-fed; females, 266.7 ± 4.4 g for chow-fed vs. 324.5 ± 6.2 g for HED-fed; *p* < 0.001). There were also significant differences in percent body fat with main effects of sex (F (1, 44) = 6.404, *p* = 0.015), and diet (F (1, 44) = 169.3, *p* < 0.001) but no significant interaction (F (1, 44) = 0.831, *p* = 0.367). Chow-fed males had a significantly lower proportion of body fat than HED-fed males (mean percent body fat = 79.01 ± 0.67% for chow fed vs. 23.27 ± 1.52% for HED-fed; *p* < 0.001). The same was true for females (mean percent body fat = 7.35 ± 0.4% for chow-fed vs. 19.74 ± 1.13% for HED-fed, *p* < 0.001) (Figure 1A). Diet had no significant effect on lean body mass (F (1, 44) = 0.813, *p* = 0.372); however there was a significant effect of sex, with males having greater body mass that females in general (F (1, 44) = 548.8, *p* < 0.001) (Figure 1A). In sum, rats exposed to a HED for 10 weeks weighed more than rats exposed to a chow diet, with the difference largely accounted for by increases in the percent body fat. Figure 1B shows examples of the results of DXA scans of male and female rats that were fed either chow or an HED for 10 weeks.

### 3.2. Effect of Microglia Suppression on Body Weight Was Sex- and Diet-Dependent

To investigate the role of microglial suppression on body weight and composition during different stages of obesity, PLX-adulterated diets were presented to chow-fed rats (Chow/Chow+PLX), to chow-fed rats being introduced to a HED after 10 weeks on chow (Chow/HED+PLX), and to rats after 10 weeks of HED consumption (HED/HED+PLX). Body weights and food consumption were averaged at the end of each week of exposure to PLX-adulterated chow or HEDs, as well as HED or chow diet in separate groups.

**Figure 2:**
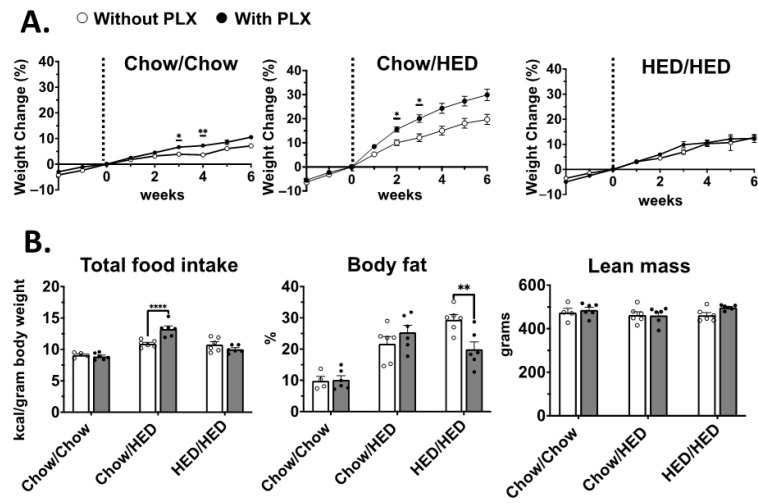
Male: Length of HED exposure dictates the effect of PLX on body weight, body fat and food intake. (**A**). Body weight gained as a percentage relative to the weight at the start of PLX consumption; vertical dashed line indicates the start of the second diet for each dietary condition. Left: Graph shows a significant difference in percentage of weight gain during weeks 3 (*p* < 0.05) and 4 (*p* < 0.01) on the Chow/Chow+PLX vs. Chow/Chow diet. Middle: graph shows a significant difference in percentage of weight gain during weeks 2 (*p* < 0.05) and 3 (*p* < 0.05) on the Chow/HED+PLX vs. Chow/HED. Right: PLX did not change the weight gain trajectory in HED/HED+PLX vs. HED/HED groups. (**B**). Left: cumulative food intake normalized for body weight. Food intake increased in Chow/HED+PLX vs. Chow/HED rats (*p* < 0.0001). Middle: Final percent body fat in all groups. The addition of PLX to the HED significantly reduced body fat in the HED/HED+PLX vs. HED/HED rats (*p* < 0.01) Right: Addition of PLX to the diet did not affect lean mass in any group. Data are expressed as mean ± SEM. * *p* < 0.05, ** *p* < 0.01, **** *p* < 0.0001. All statistics performed using two-way ANOVA with Šidák correction for multiple comparisons.

For male rats, the addition of PLX to the diet paradoxically tended to increase body weight, rather than reduce it. For example, presentation of the PLX-adulterated diet significantly increased body weight in Chow/Chow+PLX rats (Figure 2A, left, effect of diet, F (1, 8) = 13.73, *p* = 0.007; effect of time, F (3.26, 26.1) = 188.1, *p* < 0.001; interaction, F (8, 64) = 1.481). Comparisons of individual weeks showed significant differences in weeks 3 (*p* = 0.02) and 4 (*p* = 0.008). This effect could not be accounted for by changes in food intake (Figure 2B, left; *p* > 0.999). Similarly, PLX increased body weights in Chow/HED vs. Chow/HED+PLX rats (Figure 2A, middle, effect of diet, F (1, 10) = 14.0, *p* = 0.004, effect of time, F (1.5, 15) = 226.0, *p* < 0.001; interaction, F (7, 70) = 6.4, *p* < 0.001). Mean body weights were significantly different for weeks 2 (*p* = 0.05) and 3 (*p* = 0.044). Food consumption was significantly increased in Chow/HED+PLX compared with Chow/Chow rats (Figure 2B, left; F (2, 28) = 12.30, *p* < 0.0001). For the comparison of HED/HED vs. HED/HED+PLX groups (Figure 2A, right) there was a significant effect of time (F (2.02, 20.17) = 78.08, *p* < 0.001) but no significant effect of diet (F (1, 10) = 0.208, *p* = 0.658) and no significant interaction (F (7, 70) = 1.062, *p* = 0.397). Microglial suppression produced distinct body-weight trajectories between sexes: males tended to gain weight, whereas females often maintained or lost weight under PLX treatment. These opposite outcomes parallel known protective effects of estrogen on central microglial activation and inflammatory signaling. However, there was a significant decrease in the percent of body fat (Figure 2B, middle; F (2, 28) = 9.20, *p* = 0.0009). There were no changes in lean mass in any diet condition (Figure 2B, right; *p* > 0.1). Overall, the addition of PLX to the diet, either Chow or when the HED was first introduced, produced an increase in body weight. When the rats were on the HED for 10 weeks, the subsequent addition of PLX decreased the percent body fat without either increasing or decreasing body weights.

**Figure 3:**
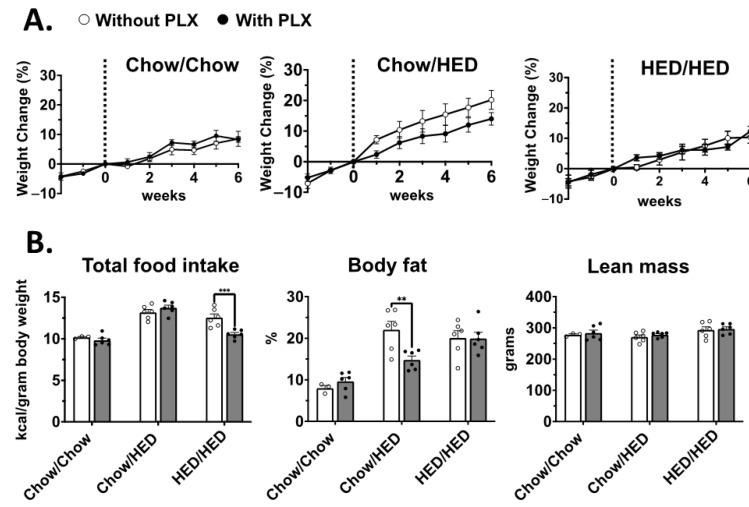
Female: PLX decreases body weight and body fat depending on length of HED exposure. (**A**). Body weight gained as a percentage relative to the weight at the start of PLX consumption; vertical dashed line indicates the start of the second diet for each dietary condition. Addition of PLX to the diet did not significantly alter weight gain trajectory in any group. (**B**). Left: Cumulative food intake normalized for body weight. Rats in the HED/HED+PLX group at significantly less than rats in the HED/HED group. Middle: Final percent body fat in all groups. The addition of PLX to HED resulted in a significant decrease in body fat percentage (*p* < 0.01) for the Chow/HED+PLX vs. Chow/HED+PLX groups. Right: Addition of PLX to the diet did not affect lean mass in any group. Data are expressed as mean ± SEM. ** *p* < 0.01, *** *p* < 0.001. All statistics performed using two-way ANOVA with Šidák’s correction for multiple comparisons.

For females there were no significant effects of PLX consumption on body weight (Figure 3A), though Chow/HED-fed females did trend toward a decrease in body weight (Figure 3A, middle). HED/HED+PLX females ate significantly less (Figure 3B, left; F (1, 27) = 4.88, *p* = 0.0358) than HED/HED rats, while Chow/HED+PLX females showed significantly decreased body fat percentages (Figure 3B, middle; F (2, 27) = 3.541, *p* = 0.004) with no changes to lean mass in any diet condition (Figure 3B, right). Substantial individual variability was observed in both baseline susceptibility to diet-induced obesity and responsiveness to PLX. This heterogeneity was especially pronounced in males under prolonged HED exposure, suggesting potential genotype- or hormone-related modulation of microglial reactivity. In conclusion, PLX diet adulteration had little effect on female body weight; however, PLX did reduce the percent body fat but only when introduced along with the HED.

### 3.3. Sucrose Preference

**Figure 4:**
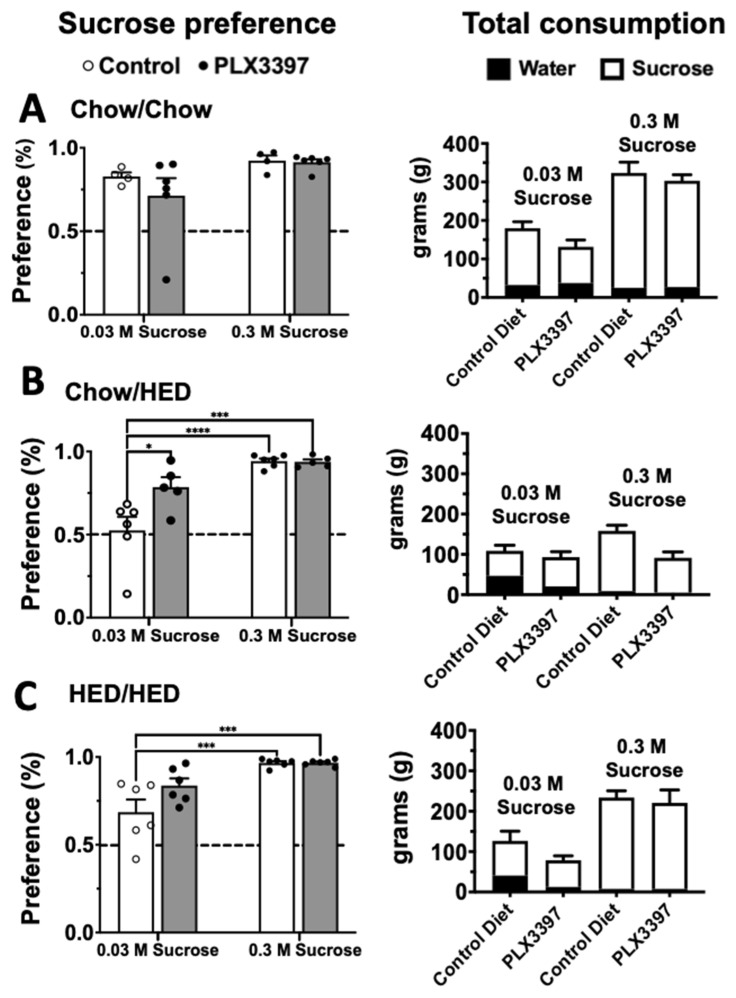
Males: Microglial suppression alters sucrose preference in male rats. Left column: 2-bottle sucrose preference tests for low (0.03 M) and high (0.3 M) concentrations of sucrose vs. tap water for each diet condition (**A**) Chow/Chow, (**B**) Chow/HED and (**C**) HED/HED. Corresponding total consumption is shown to the right of each graph. Testing was performed following the last DXA scan. Each concentration was tested for two days with bottle position switched between day 1 and 2. Bottles were weighed twice per day and never allowed to be less than 50% full. Data are expressed as mean ± SEM. * *p* < 0.05, *** *p* < 0.001, **** *p* < 0.0001. All statistics performed using two-way ANOVA with Šidák’s correction for multiple comparisons.

There is evidence that acute microglial ablation in male rats leads to pronounced aversion to sweet tastes [5]. We sought to determine if chronic suppression of microglial had the same effect. To test this, we used a two-bottle sucrose preference test with two concentrations of sucrose, 0.03 M and 0.3 M. Preference scores were analyzed using a two-way ANOVA. For males, results showed a significant effect of diet group (F (5, 54) = 2.595, *p* = 0.036), sucrose concentration (F (1, 54) = 46.38, *p* < 0.001) and a significant diet group-sucrose concentration interaction (F (5, 54) = 2.498, *p* = 0.042). Pairwise individual comparisons (using a Tukey correction for multiple comparisons) showed that six weeks of a HED in the Chow/HED group reduced preference for 0.03 M sucrose to near chance level while adulteration with PLX prevented this suppression and essentially restored preference to control levels (Figure 4B). These results indicate that the restoration of sucrose preference in PLX-treated animals likely reflects normalization of hedonic sensitivity rather than nonspecific motivational changes. On the other hand, 16 weeks on a HED in the HED/HED group had no effect on preference for 0.03 M sucrose, suggesting that the suppression of 0.03 M sucrose preference was temporary. Male rats across all diet groups including those with PLX adulteration exhibited a robust preference for 0.3 M sucrose (Figure 4). Analogous analyses for females showed no significant differences in sucrose preference for any diet group (*p* > 0.1), though there was a significant effect of sucrose concentration (F (1, 54) = 23.86, *p* < 0.001). That is, all female rats preferred 0.3 M sucrose significantly more than 0.03 M sucrose. In all, exposure to the HED for six weeks in male rats reduced sucrose preference but adulteration of the HED with PLX prevented this reduction, but neither effect was present in female rats.

**Figure 5:**
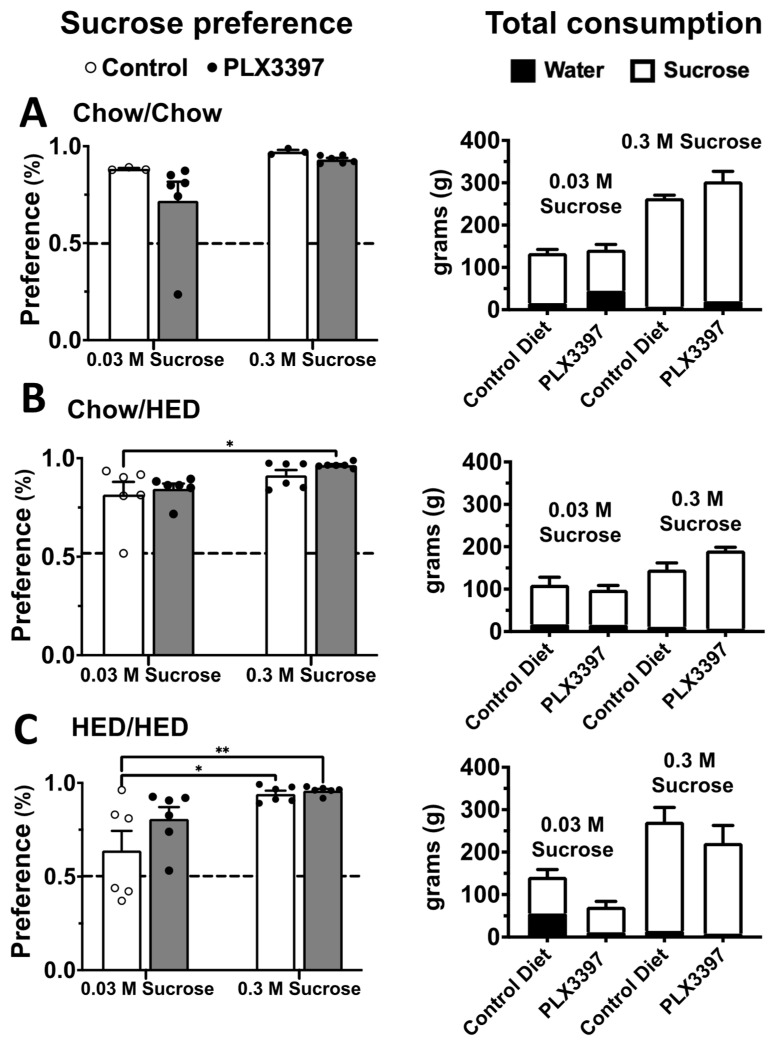
Females: Microglial ablation alters sucrose preference in female rats. Left column: 2-bottle sucrose preference tests for low (0.03 M) and high (0.3 M) concentrations of sucrose vs. tap water for each diet condition (**A**) Chow/Chow, (**B**) Chow/HED and (**C**) HED/HED. Corresponding total consumption is shown to the right of each graph. Testing was performed following the last DXA scan. Each concentration was tested for two days with bottle position switched between day 1 and 2. Bottles were weighed twice per day and never allowed to be less than 50% full. Data are expressed as mean ± SEM. * *p* < 0.05, ** *p* < 0.01. All statistics performed using two-way ANOVA with Šidák’s correction for multiple comparisons.

The effects of diet and PLX adulteration on total liquid consumption (water plus sucrose) revealed additional effects of the HED and PLX-adulterated diet (see Figure 5). In male rats, a two-way ANOVA of total consumption showed a significant effect of diet group (F (5, 54) = 11.64, *p* < 0.001) and sucrose concentration (F (1, 54) = 25.37, *p* < 0.001) as well as a significant diet group-sucrose concentration interaction (F (5, 54) = 15.47, *p* < 0.001). When 0.03 M sucrose was presented along with water, the HED/HED+PLX group drank significantly more fluid compared to all other groups except for the Chow/Chow group (*p* < 0.05; Tukey correction for multiple comparisons). Notably, although PLX adulteration restored sucrose preference in Chow/HED+PLX vs. Chow/HED rats, it had no effect on total fluid consumption. When 0.3 M sucrose was presented along with water, fluid consumption was nearly universally lower in groups that were exposed to a HED (*p* < 0.01) although sucrose preference was near total in all diet groups. PLX adulteration significantly suppressed fluid consumption only in the HED/HED+PLX group (*p* < 0.001). For females, two-way ANOVA showed a significant effect of diet group (F (5, 54) = 6.420, *p* < 0.001) and sucrose concentration (F (1, 54) = 75.37, *p* < 0.001) but no significant interaction (F (5, 54) = 2.268, *p* = 0.061). There were no significant differences among any diet groups for 0.03 M sucrose where total fluid consumption was generally lower than that for 0.03 M sucrose. Also, as with male rats, diet groups exposed to a HED generally drank less total fluid than the Chow/Chow or Chow/Chow+PLX groups, but sucrose preference was unaffected.

### 3.4. PLX Suppression of Microglia Activation in the Hypothalamus

To examine PLX suppression of microglia activation in the hypothalamic nuclei, we quantified the number of activated microglia in male (*n* = 30) and female (*n* = 27) rats across diet groups. Initially, we counted activated microglia separately in the left and right arcuate nucleus (Arc), the paraventricular nucleus (PaVN), the lateral hypothalamic nucleus (LH), the ventromedial nucleus (VMH) and the dorsomedial nucleus (DMN). We also examined the periventricular nucleus (PeVN) and the median eminence (ME) in both sexes across diet groups. The only significant difference in microglia expression on the left vs. right sides was found in the male LH (F (1, 40) = 4.245, *p* = 0.046) where the right LH showed more microglial expression than the left. In all other hypothalamic nuclei, there were no significant effects of side. For further analyses, we pooled the numbers from left and right sides to assess the effects of diet group and sex. Two-way ANOVAs were performed with sex and diet group as main factors. The results are shown in Table 1 and Figure 5. There were no significant effects of either diet group or sex in the PeVN.

**Figure 6:**
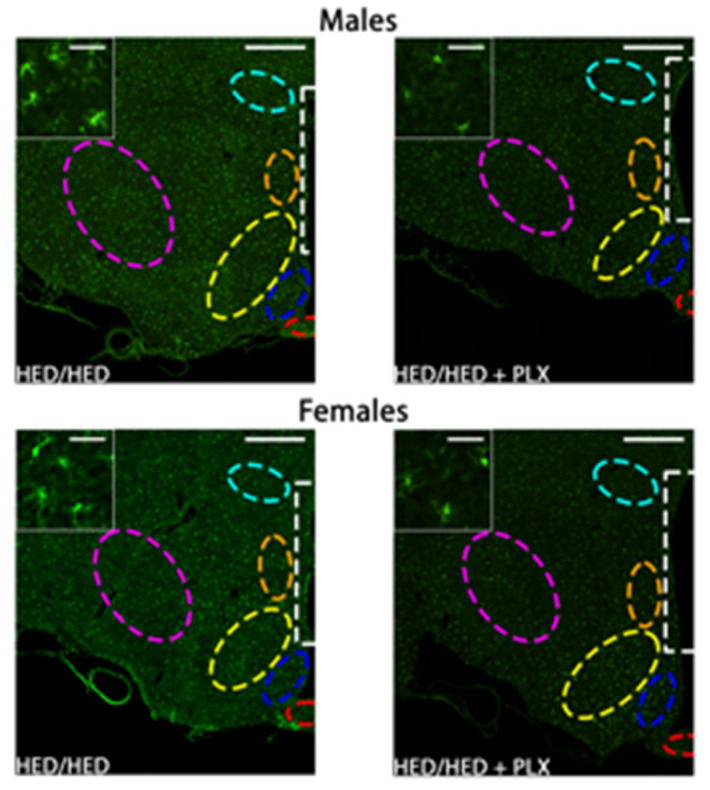
Effects of PLX diet adulteration on microglial activation in the hypothalamus. Representative coronal 20× images of Iba1 immunoreactivity in the hypothalamus near Bregma −1.92 mm in males and females. Inserts are 120× images from the same section demonstrating microglial morphology with each of the diet and treatment combinations. Male and female HED/HED and HED/HED + PLX inserts are from the dorsomedial nucleus. Regions of interest (ROIs) are red = median eminence, blue = arcuate nucleus, yellow = ventromedial nucleus, orange = dorsomedial nucleus, white = periventricular nucleus, light blue = paraventricular nucleus, pink = lateral hypothalamic area.

**Figure 7:**
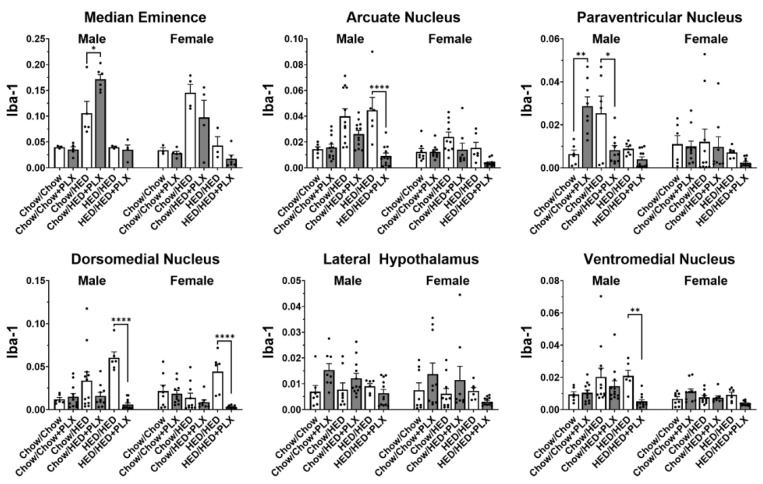
Results of analyses of quantitative measures of Iba1 immunoreactivity in hypothalamic nuclei. Data from males and females were analyzed separately. * *p* < 0.05, ** *p* < 0.01, **** *p* < 0.0001. All statistics were performed using a two-way ANOVA with Tukey’s correction for multiple comparisons.

Analyses of microglia expression showed that the degree of microglial activation in the hypothalamus varied with both diet group and sex depending on the particular nucleus (see Figure 6 and Figure 7). Exposure to a HED for 10 weeks significantly increased activated microglial density only in the Arc of male rats (Chow/HED group). In contrast, it took 16 weeks of a HED to significantly increase activated microglial density in the DMN of male and the ME of females (HED/HED group). The addition of PLX to the HED (HED/HED vs. HED/HED+PLX groups) significantly decreased microglial activation in the Arc, VMH and DMN in male rats and in the DMN of female rats, but only after they had already been on the HED for 10 weeks. In the ME of male rats, the addition of PLX to the diet significantly increased microglial activation, perhaps reflecting the body weight increase correlated with PLX diet exposure in male rats (see Figure 2). PLX produced robust suppression of Iba1+ microglia in both hypothalamus and NTS. However, the relative magnitude of suppression differed by region, supporting the idea that PLX may differentially affect microglial subpopulations depending on local CSF-1R expression and diet context. In sum, results show that the pattern of microglial expression in the hypothalamic nuclei differs in males vs. females, with less expression across the board in the hypothalamus of females with males regardless of diet group compared (*t* = 3.485, df = 379). Not surprisingly, microglial proliferation in the hypothalamus of both males and females is greatest when animals are exposed to a HED for longer periods of time. Moreover, the addition of PLX to the diet had the greatest effect on activated microglial density in those animals that have had HED for 10 weeks but does not prevent microglial proliferation when given along with the HED.

### 3.5. PLX Suppression of Microglia Activation in the NTS

**Figure 8:**
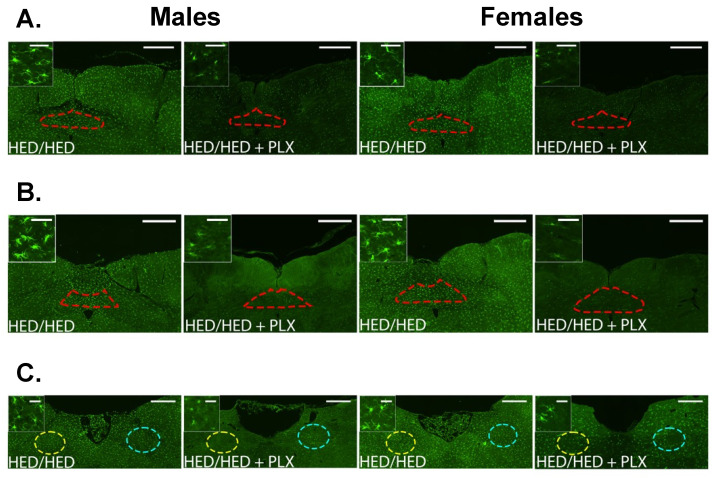
PLX Suppresses microglia activation in the caudal, intermediate, and rostral NTS. (**A**–**C**) Representative 20× images of Iba1 immunoreactivity in the caudal (**A**), intermediate (**B**), and rostral (**C**) NTS near Bregma −14.52 mm, −14.04 mm, and −12.6 mm, respectively. Inserts are 120× images from the same section demonstrating microglial morphology with each of the diet and treatment combinations. Regions of interest (ROIs) are red in the caudal and intermediate NTS (**A**,**B**) and are divided into left and right, yellow, and blue in the rostral NTS (**C**).

**Figure 9:**
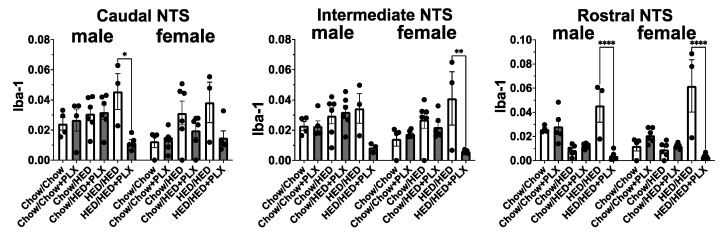
Results of analyses of quantitative measures of Iba1 immunoreactivity in the NTS. Data from males and females were analyzed separately. * *p* < 0.05, ** *p* < 0.01, **** *p* < 0.0001. All statistics were performed using a two-way ANOVA with Tukey’s correction for multiple comparisons.

By including both the hypothalamus and NTS, our study reveals that microglial modulation within these two regions contributes differently to metabolic and hedonic outcomes. Hypothalamic microglial depletion primarily affected body-fat regulation, whereas NTS alterations were more closely linked to sucrose preference and reward signaling. These findings reinforce the concept that hypothalamic and brainstem circuits exert complementary, region-specific control over feeding and taste behavior. As predicted, PLX significantly suppressed microglia activation in all three regions of the NTS (rostral, intermediate, and caudal) in rats that had been fed HED (see Figure 8 and Figure 9). As with the hypothalamus, we first analyzed potential differences in activated microglial density between the left and right caudal, intermediate and rostral NTS in male and female rats using a two-way ANOVA. Results showed that in all three regions of the NTS, there were no significant differences (*p* > 0.5). Given that result, we pooled data from left and right sides in each area to then analyze diet group effects in male and female rats, also using a two-way ANOVA with sex and diet group as factors. Results are shown in Figure 8. There were no significant differences between male and female rats for the intermediate and rostral NTS (*p* > 0.2). However, the effect of sex was nearly significant (F (1, 46) = 3.86, *p* = 0.056) in the caudal NTS, but not quite. For all three areas of NTS, there were significant effects of diet group (*p* < 0.001). There were no increases in microglia expression after six weeks on the HED (Chow/HED group) even though these rats showed a significant increase in the percent body fat over their Chow/Chow counterparts (see Figure 3). In contrast, rats in the HED/HED groups showed evidence of significant microglia proliferation which was significantly reduced by the addition of PLX to the diet in most rats. In all, microglia in the NTS do not show signs of proliferation until the animal has been on the HED for a prolonged period (~16 weeks). The addition of PLX to a HED suppressed activated microglia density only when the animal had already been on a HED for 10 weeks.

## 4. Discussion

Microglia are well established regulators of energy homeostasis and susceptibility to DIO via inflammatory signaling in both obese and healthy physiological states. Microgliosis in several brain regions central to regulation of feeding behavior leads to overconsumption and subsequent obesity pathology. However, previous studies are equivocal about sexual dimorphisms in both rat and mouse models [5,11,28]. In the present study, we show sexually dimorphic effects of depletion of microglia on body weight, body composition and sucrose preference. These results correlate with activated microglial density changes in NTS and hypothalamic structures following PLX administration. However, variability in the effectiveness of PLX both in affecting body weight and composition and in affecting activated brain microglial density underscores the importance of individual differences in susceptibility to obesity and effectiveness of potential treatments. Although PLX3397 is primarily used as a CSF-1R antagonist to suppress microglia, it can also inhibit other receptor tyrosine kinases such as c-Kit and FLT3 [29,30,31]. These off-target effects may influence peripheral immune cells or metabolic pathways and could partially contribute to the sex-specific responses observed in this study. Notably, individual variability in weight trajectories and sucrose preference responses suggests that baseline microglial tone and hormonal environment strongly influence treatment outcomes. Such variability underscores the need for individualized assessment in translational applications of microglial modulation. Nevertheless, these data show that microglial depletion can reduce body weight in subjects with obesity.

**Figure 10:**
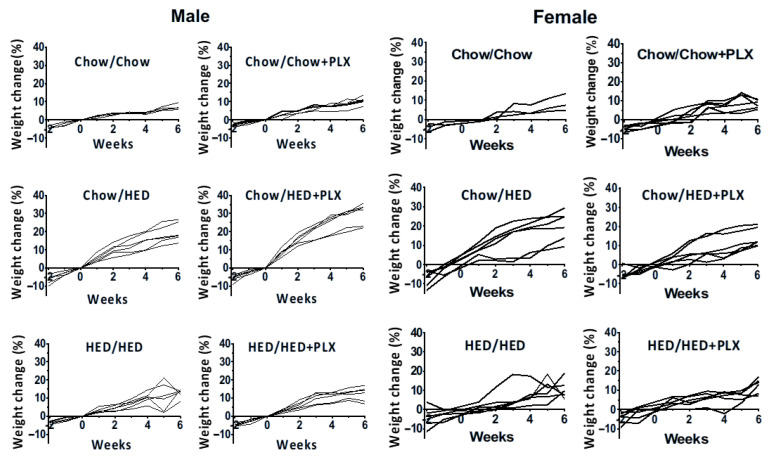
Individual differences in the effects of PLX on weight gain in male and female rats. Graphs show percent weight gain with respect to baseline weight at 0 weeks for each subject in each group.

Although the effects of the HED on body composition were as expected, that is all rats had increased body weight and especially percent body fat, the effects of the addition of PLX to the diet were highly variable across subjects (see Figure 10). For example, for male rats in the Chow/HED group exposed to the HED for 6 weeks, HED+PLX significantly and unexpectedly enhanced weight gain in four of six rats, while two rats showed no effect of PLX. As a group, there was no effect of PLX on percent body fat in Chow/HED male rats. In contrast, all rats in the HED/HED and HED/HEDPLX groups gained weight but HED adulteration with PLX resulted in a significant decrease in body fat percentage, though not to the level shown by lean rats. In female Chow/HED rats, two of six rats did not gain weight on the HED. In the Chow/HED+PLX group, four of six rats showed weight trajectories that resembled those in the Chow/Chow group, suggesting that the PLX-adulterated HED prevented the development of obesity to varying degrees depending on the individual. However, the weight gain in the two rats that were apparently unaffected by HED+PLX likely prevented group effects from attaining statistical significance. For female rats in the HED/HED and HED/HED+PLX groups, there was consistently no effect of PLX on either body weight or percent body fat. Considered together, these data may reflect the well-described characteristics of DIO resistance and DIO susceptibility within many rat strains, including Sprague-Dawley [32,33,34].

Our data showed enhanced PLX effectiveness in reducing activated microglial density and changing body compositions when the rats had already been exposed to a HED for 10 weeks. One primary consequence of HED consumption is the development of insulin resistance [35], and recently the meta-inflammatory nature of obesity is seen as the focal point for insulin resistance [36]. Mechanisms responsible for insulin resistance have been shown to vary over a 10-week HED period; lipid overload and lipo-toxicity are critical factors early on while systematic inflammation becomes relevant later [37]. Further, central inflammation is highly dependent on brain region and time-dependent, with different brain regions having variable inflammatory profiles at several intervals between 1 and 16 weeks [38]. Our findings recapitulate these conclusions and suggest a close longitudinal relationship between central inflammation and exposure to HEDs.

Data on the role that microglia play in satiety and metabolism is contradictory. Microglial inactivation has been shown to have a suppressive effect on food intake and body weight [5,9], while microglia activation has also shown similar results [39]. Several methodological factors must be considered when interpreting consummatory behavior following microglial inhibition or ablation. In mice, visfatin-induced microglia activation leads to decreased food intake and anorexia, and microglial inhibition with minocycline rescues this effect [8]. Oral consumption of PLX5662, a more brain-penetrant CSF-1R inhibitor than PLX3397, produces no alterations in consummatory behavior or weight gain in mice, while genetic activation of microglia by tumor necrosis factor a-induced protein 3 (TNFAIP3) causes obesity [9]. In contrast, acute genetic ablation of microglia in chow-fed male rats causes anorexia [5], whereas we show chronic pharmacologic ablation leads to weight gain at least in male rats. Obviously, more work is needed to clarify the sources of methodological variability among microglial ablation techniques.

Our findings suggest that microglial modulation may hold translational potential for obesity prevention and treatment. Inhibition of CSF-1R signaling with PLX3397 altered body weight and sucrose preference, supporting the role of microglia in both metabolic regulation and reward-driven feeding. Because hypothalamic microglial activation contributes to diet-induced obesity, selective suppression of this neuroinflammatory response could mitigate metabolic dysfunction. Nevertheless, microglia are essential for neuronal homeostasis and synaptic remodeling; thus, chronic or global inhibition may be detrimental. Future therapeutic strategies should aim to fine-tune microglial activity, targeting proinflammatory pathways or employing temporal control, to restore normal energy balance while preserving central nervous system function.

Although PLX3397 targets CSF-1R and thereby depletes microglia, potential off-target effects on peripheral macrophage populations cannot be completely excluded. However, control studies in rodents and primates indicate that PLX at similar doses selectively reduce Iba1+ microglia with minimal systemic immune alteration. Our interpretation therefore focuses on central, rather than peripheral, mechanisms.

### 4.1. Microglia in the Brain

Rapidly growing evidence suggests that hypothalamic microglia mediate obesity susceptibility. As with body weight and composition, the effectiveness of the HED in producing a significant increase in activated microglia, especially in the hypothalamus, and the effectiveness of the PLX-adulterated diet in reducing microglial density were highly variable across subjects. This may, in part, account for the lack of group effects where these effects were to be expected. For example, the consumption of a HED was expected to induce microglia proliferation in the hypothalamus, but this was observed only inconsistently across nuclei. Furthermore, where HED-associated microglia proliferation was observed, the adulteration of the diet with PLX ameliorated the proliferation only in a subset of hypothalamic nuclei and then not always to a statistically significant degree. Nevertheless, there were clear sex differences not only in the overall microglia density (males showed higher density of activated microglia than females) and in the effects of an HED and an HED adulterated with PLX, where PLX was generally more effective in males than in females. This may reflect a floor effect.

Systemic obesity-related inflammation has recently been described as longitudinal, starting with gut microbiota dysbiosis followed by CNS microglial activation [40]. Peripheral inflammatory markers are relayed to the NTS via the vagus nerve, a pathway also responsible for meditating consummatory behavior [41]. Vagal-NTS synapses are plastic and will reorganize in the presence of HED consumption, leading to NTS inflammation and associated weight gain [24]. In the present study, significant NTS microglial activation was observed in the prolonged presence of a HED, which agrees with prior studies [24]. The addition of PLX to the HED reduced the density of microglia at all levels of the NTS in both male and female rats. This evidence underscores the complex interaction between HED consumption and inflammation, with the NTS as a critical node for consummatory and possibly inflammatory signal transfer. The NTS has additionally been shown to transfer both orexigenic and anorexigenic signals to upstream brain structures including the hypothalamus [42,43,44], thus further research is needed to precisely segregate the modulatory capabilities of NTS neurons and microglia.

Future studies should incorporate additional behavioral paradigms, including affective and operant assays such as forced swim, novelty, suppressed feeding, and progressive ratio tasks, to more fully disentangle hedonic, motivational, and sensory components of sucrose preference following microglial modulation.

### 4.2. Sex Differences

To further clarify the mechanistic basis of these sex-dependent effects, it is important to note that estradiol and other ovarian hormones are known to exert neuroprotective and anti-inflammatory actions on microglia [PMID: 18460549, 11014219, 16013043]. Reduced activation of hypothalamic and NTS microglia in females could thus contribute to their relative resistance to PLX-induced metabolic disruption. Conversely, testosterone-associated enhancement of inflammatory signaling in males may sensitize microglia to PLX-mediated depletion and secondary metabolic effects.

Although the effects of the HED on body composition were as expected, that in all rats increased body weight and especially percent body fat, the effects of the addition of PLX to the diet were highly variable and sex-specific (see Figure 8 and Figure 9). Surprisingly, when PLX was consumed along with the HED, male rats, but not females, gained weight without changes in body fat. Specifically, four of six male rats in the Chow/HED+PLX group gained about 30% of their pre-HED weight. This weight gain was about 10% more than the rats gained in the group without PLX (Chow/HED). However, two rats gained approximately the same weight as rats in the Chow/HED group, suggesting that PLX diet adulteration either had no effect or was protective in these rats. As a group, there was no effect of PLX on percent body fat in Chow/HED male rats. In contrast, all rats in the HED/HED and HED/HEDPLX groups gained weight but HED adulteration with PLX resulted in a significant decrease in body fat percentage, though not to the level shown by lean rats. In female rats, the Chow/HED and Chow/HED+PLX groups showed obvious variability in both the effect of a HED and the effect of the addition of PLX on weight change. In the Chow/HED group, two of the six rats clearly did not gain weight with the HED, suggesting that they were obesity resistant. In the Chow/HED+PLX group, four of six female rats showed weight gain trajectories similar to those shown by Chow/Chow rats, indicating that PLX added to the diet prevented the development of obesity. However, the weight gain in the two rats in the Chow/HED+PLX group that were apparently unaffected by PLX likely prevented group effects from attaining statistical significance. For female rats in the HED/HED and HED/HED+PLX groups, there was consistently no effect of PLX on either body weight or percent body fat. Considered together, these data may reflect the well-described characteristics of DIO resistance and DIO susceptibility within many rat strains, including Sprague-Dawley [32,33,34]. In addition, these data underscore the importance of examining individual differences in obesity resistance and in the effects on weight and body composition of manipulating microglia.

Compared to males, females are generally less susceptible to diet-induced obesity [45], possibly due to anti-inflammatory sex hormones. Indeed, females showed less overall activated microglial density than males in the present study. Data from humans provides some hints at underlying mechanisms that may contribute to these apparent sex differences. For example, premenopausal women are protected from the metabolic consequences of obesity, effects that return post menopause [46]. 17b-estradiol (E2), the major female sex hormone, is present in males but in much lower levels than females and has been shown to have strong anti-inflammatory properties [46,47,48]. Preovulatory increases in estradiol secretion have been associated with decreased food intake in rats, and silencing the E2-receptor in mice and rats leads to increased food intake, glucose intolerance, hyperphagia and obesity [49]. E2 and its receptor, E2R, inhibit lipopolysaccharide (LPS)-induced microglial activation, suppressing proinflammatory cytokines and promoting anti-inflammatory cytokines [46,48,50]. Additionally, work by Dorfman et al. [51] suggests that for males, HED-induced microglial activation may be a compensatory response to blunted CX3CL1-CX3CR1 signaling and reduced pro-opiomelanocortin (POMC) neuron activation in the hypothalamus, while females retain this interaction and are subsequently protected from DIO [48,52].

## 5. Conclusions

In conclusion, this study demonstrates that microglial ablation with the CSF1R antagonist PLX3397 exerts sex- and diet-dependent effects on body weight, body composition, and consummatory behavior. In males, PLX treatment increased body weight under chow and HED conditions but reduced adiposity in diet-induced obesity (DIO), whereas in females it decreased body weight and fat mass only under chow feeding, with minimal effects in DIO animals. These metabolic outcomes were accompanied by suppression of HED-induced microglial proliferation in both the hypothalamus and the nucleus tractus solitarius (NTS), identifying the NTS as a relevant yet underexplored site of neuroinflammatory regulation in obesity. Females exhibited lower baseline microglial activation but remained sensitive to PLX-mediated modulation within specific hypothalamic and brainstem regions. Behavioral effects were modest, with PLX partially restoring sucrose preference in chow- and HED-fed males. Future studies including correlative analyses of microglial density and phenotype with behavioral and metabolic outcomes would allow us to better delineate individual variability in drug response.

Overall, our findings highlight pronounced sexual dimorphism in neuroimmune and metabolic responses to high-energy diets, suggesting relative female resilience and male vulnerability to diet-induced neuroinflammation. These results underscore the importance of incorporating sex as a biological variable in studies of metabolic regulation and support the potential of targeted microglial modulation as a therapeutic strategy to mitigate neuroinflammation and restore energy balance in obesity. Our results do not imply a universally beneficial or detrimental role of microglial suppression. Instead, they suggest that microglia participate in fine-tuning both metabolic and motivational systems in a context-dependent manner, modulated by diet and sex hormones.

## Figures and Tables

**Table 1:** Results of two-way ANOVA of microglia expression in the various subregions of the hypothalamus and NTS in male and female rats across diet groups. * *p* < 0.05; ** *p* < 0.01; *** *p* < 0.001.

Structure	Diet Group	Sex	Interaction
Arc	F (5, 45) = 9.73, *p* < 0.001 ***	F (1, 45) = 16.83, *p* < 0.001 ***	F (5, 45) = 1.89, *p* = 0.011 *
PaVN	F (5, 39) = 3.67, *p* = 0.008 **	F (1, 39) = 2.38, *p* = 0.131	F (5, 39) = 1.68, *p* = 0.162
LH	F (5, 40) = 4.82, *p* = 0.002 **	F (1, 40) = 1.25, *p* = 0.271	F (5, 40) = 0.35, *p* = 0.880
VMH	F (5, 45) = 2.9, *p* = 0.024 *	F (1, 45) = 9.06, *p* = 0.004 **	F (5, 45) = 1.47, *p* = 0.217
DMN	F (5, 44) = 6.77, *p* < 0.001 ***	F (1, 44) = 1.38, *p* = 0.247	F (5, 44) = 0.93, *p* = 0.470
ME	F (5, 39) = 24.88, *p* < 0.001 ***	F (1, 39) = 1.44, *p* = 0.237	F (5, 39) = 3.49, *p* = 0.011 *
PeVN	F (5, 45) = 1.51, *p* = 0.205	F (1, 45) = 8.07, *p* = 0.374	F (5, 45) = 0.46, *p* = 0.807
cNTS	F (5, 46) = 5.05, *p* < 0.001 ***	F (1, 46) = 3.86, *p* = 0.056	F (5, 46) = 0.81, *p* = 0.547
iNTS	F (5, 45) = 6.15, *p* < 0.001 ***	F (1, 45) = 1.45, *p* = 0.236	F (5, 45) = 0.51, *p* = 0.766
rNTS	F (5, 48) = 20.94, *p* < 0.001 ***	F (1, 48) = 0.08, *p* = 0.784	F (5, 48) = 1.57, *p* = 0.187

## Data Availability

The original data presented in the study are openly available in FigShare at https://doi.org/10.6084/m9.figshare.21420462.v1 (accessed on 27 October 2022).

## Abbreviations

Arc	Arcuate nucleus
Atip	Atipamezole
CNS	Central nervous system
CSF-1R	Colony-stimulating factor 1 receptor
DIO	Diet-induced obesity
DMN	Dorsomedial nucleus of hypothalamus
DXA	Dual-energy X-ray absorptiometry
HED	High-energy diet
iNTS	Intermediate nucleus tractus solitarius
i.p.	Intraperitoneal
LH	Lateral hypothalamus
Med	Medetomidine hydrochloride
ME	Median eminence
NTS	Nucleus tractus solitarius
PaVN	Paraventricular nucleus of hypothalamus
PeVN	Periventricular nucleus of hypothalamus
PLX	Pexidartinib (PLX3397)
rNTS	Rostral nucleus tractus solitarius
s.c.	Subcutaneous
SD	Sprague-Dawley
VMH	Ventromedial hypothalamus
